# Use of GPT-4 to Analyze Medical Records of Patients With Extensive Investigations and Delayed Diagnosis

**DOI:** 10.1001/jamanetworkopen.2023.25000

**Published:** 2023-08-14

**Authors:** Yat-Fung Shea, Cynthia Min Yao Lee, Whitney Chin Tung Ip, Dik Wai Anderson Luk, Stephanie Sze Wing Wong

**Affiliations:** 1 Department of Medicine, Queen Mary Hospital, University of Hong Kong, Hong Kong

## Abstract

This case series investigates whether analysis of clinical history via a language model system improves diagnostic accuracy in patients with complex and delayed diagnoses.

## Introduction

Artificial intelligence (AI), especially machine learning, has been increasingly used in diagnosing conditions such as skin or breast cancer and Alzheimer disease. However, AI relies on clinical imaging.^1^ In low-income countries, where specialist care may be lacking, AI may be useful for making clinical diagnoses. The GPT-4 (Generative Pre-trained Transformer 4) program allows analysis of clinical history in daily practice.^2^ We hypothesized that GPT-4 could improve the diagnostic accuracy of clinicians by supplying the most probable diagnosis or suggesting differential diagnoses in complex cases.

## Methods

The medical histories of 6 patients from the Division of Geriatrics in the Department of Medicine at Queen Mary Hospital who were aged 65 years or older and had delay of definitive diagnosis longer than 1 month in 2022 were retrieved after resolution.^3,4,5^ The full medical histories were entered chronologically on April 16, 2023 (at admission, 1 week after admission, and before final diagnosis) into GPT-4 (powered by OpenAI via Platform for Open Exploration) without information about definitive diagnosis. The GPT-4 responses were copied out and further analyzed (eMethods in Supplement 1). One patient has been described previously.^6^ Responses by GPT-4 and clinicians were collected and compared. Differential diagnoses were also generated using a medical diagnostic decision support systemIsabel DDx Companion; Isabel Healthcare). The study was approved by the Institutional Review Board of the University of Hong Kong and Hospital Authority Hong Kong West Cluster. Written consent was provided for all patients. This report followed the reporting guideline for case series studies.

## Results

Six patients 65 years or older (2 women and 4 men) were included in the analysis. The accuracy of the primary diagnoses made by GPT-4, clinicians, and Isabel DDx Companion was 4 of 6 patients (66.7%), 2 of 6 patients (33.3%), and 0 patients, respectively. If including differential diagnoses, the accuracy was 5 of 6 (83.3%) for GPT-4, 3 of 6 (50.0%) for clinicians, and 2 of 6 (33.3%) for Isabel DDx Companion (Table). By studying the changes in GPT-4’s responses, we determined that certain key words were required to make an appropriate clinical response, including abdominal aortic aneurysm (patient 1), proximal stiffness (patient 2), acid-fast bacilli in urine (patient 3), metronidazole (patient 4), and retroperitoneal lymphadenopathy (patient 6). GPT-4 could suggest diagnoses not considered by clinicians before definitive investigations: mycotic aneurysm for patient 1 after computed tomography showing an abdominal aortic aneurysm; a drug cause of seizure in patient 5; and the presence of necrotic lymph nodes from a previous computed tomographic scan, which should have led to the diagnosis of lymphoma, in patient 6.

**Table.** Summary of Responses From GPT-4 and Clinicians in 6 Cases of Older Patients With Diagnostic Difficulties

Patient	Final diagnosis (supportive investigation findings)	GPT-4 response			Isabel DDx Companion keywords and top 5 DDx	Clinicians’ documentation
		On presentation	1 wk After presentation	Before final diagnosis		
Patient 1	ESBL producing *Salmonella*-related aortitis (PET scan showing mycotic aneurysm and positive blood culture)	Dx: UTI; DDx: pyelonephritis, urolithiasis, musculoskeletal pain; workup suggested: urine culture, kidney ultrasonography	Dx: UTI and/or pyelonephritis; DDx: bacteremia or sepsis, intra-abdominal infection, prosthetic infection (history of hip fracture with screws and nailing); workup suggested: repeat blood culture, CT and/or MRI of abdomen and pelvis, bone scan, consultation with microbiologist	Dx: pyelonephritis due to *Enterococcus faecium*; DDx: intra-abdominal abscess, infective endocarditis, infected abdominal aortic aneurysm, osteomyelitis, drug fever; workup suggested: blood culture, echocardiogram, consultation with microbiologist, CT and/or MRI of abdomen and pelvis	Keywords: *pyrexia of unknown origin*, *abdominal aortic aneurysm*; DDx: aortic dissection, ischemic heart disease, relapsing polychondritis, upper urinary tract obstruction, liver abscess	Dx: pyelonephritis and/or evolving kidney abscess; DDx: deep-seated infection; workup suggested: whole body gallium scan (consulted vascular surgeon and microbiologist after gallium scan)
Patient 2	Polymyalgia rheumatica (good response to prednisolone treatment)	Dx: infection and/or inflammation; DDx: inflammatory arthritis (eg, rheumatoid arthritis or polymyalgia rheumatica), infection, malignant neoplasm; workup suggested: iron studies, vitamin B_12_ and folate levels, autoimmune markers (ANA, rheumatoid factor), CT of thorax to pelvis, BME if anemia workup results unremarkable, consult microbiologist	DX: pseudogout, anemia of chronic illness or folic acid deficiency; DDx: chronic fatigue syndrome, unexplained anemia, malnutrition or vitamin deficiency or age-related physiological changes (frailty); workup suggested: nutritional assessment, endoscopy, sleep study or comprehensive geriatric assessment	Dx: polymyalgia rheumatica; DDx: pseudogout, paraneoplastic syndrome, seronegative spondyloarthropathy workup suggested: temporal artery biopsy, fine needle aspiration of the parotid lesion, trial of corticosteroid	Keywords: *weakness*, *shoulder stiffness*, *hip stiffness*; DDx: osteoarthritis, polymyalgia rheumatica, rheumatoid arthritis, rotator cuff tear, frozen shoulder	Dx: polymyalgia rheumatica; DDx: malignant neoplasm; workup suggested: consultation with rheumatology for trial of corticosteroid, parotid gland biopsy (consulted rheumatologist after geriatrician’s assessment)
Patient 3	Mycobacterium tuberculosis–related hemophagocytic lymphohistiocytosis (acid-fast bacilli in bone marrow smear and positive urine culture findings)	Dx: No primary given; DDx: CHF, liver disease, kidney disease, autoimmune or connective tissue disease; workup suggested: echocardiogram, abdominal ultrasonography (including kidney), viral hepatitis serology, autoimmune liver markers, drugs review, urinalysis, urine ratio of protein to creatinine, ANA, ANCA, complement levels	Dx: No primary given; DDx: hypoalbuminemia-related complications, autoimmune and/or connective tissue disease, myelodysplastic syndrome or other bone marrow disorder, paraneoplastic syndrome workup suggested: BME. CT of thorax to pelvis, serum protein electrophoresis and immunofixation, consult rheumatology	Dx: Disseminated tuberculosis; DDx: connective tissue disease, hematological malignant neoplasm, paraneoplastic syndrome, liver cirrhosis, other systemic infection; workup suggested: whole-body ^18^FDG-PET and/or gallium scan, bone marrow examination, autoimmune markers, CT of thorax, consultation with microbiologist, rheumatologist, and/or hematologist	Keywords: *lower limb weakness*, *thrombocytopenia*, *abnormal liver function tests*, *acid-fast bacilli positive*; DDx: thrombotic thrombocytopenic purpura, brain neoplasms, fluke infection, Tsutsugamashi disease, viral hepatitis	Dx: Mycobacterium tuberculosis–related hemophagocytic lymphohistiocytosis; DDx: malignant neoplasm; workup suggested: acid-fast bacilli staining in the trephine biopsy, whole-body ^18^FDG-PET and/or gallium scan (microbiologist had been consulted and suggested starting antituberculosis treatment after acid-fast bacilli staining in the trephine biopsy specimen was positive)
Patient 4	Chronic prostatitis (gallium scan with positive prostate uptake and good response to prolonged fosfomycin treatment)	Dx: recurrence of *E coli* bacteremia; DDx: catheter-related bloodstream infection, UTI, intra-abdominal infection, pneumonia; workup suggested: blood culture, urine culture, CT or ultrasonography of the abdomen, repeated thoracic imaging (eg, chest radiography or CT)	Dx: Recurrent ESBL *E coli* bacteremia; DDx: UTI, catheter-related bloodstream infection, endovascular infection; workup suggested: evaluate urinary tract using ultrasonography or CT, evaluate intravascular devices, CT of the abdomen, transesophageal echocardiogram	Dx: recurrent ESBL *E coli* bacteremia; no specific cause mentioned; DDx: catheter-related, infective endocarditis, osteomyelitis and/or septic arthritis, intra-abdominal infection, prostatitis, soft tissue infection, immunodeficiency; workup suggested: thorough physical examination, CT of the abdomen and pelvis, transesophageal echocardiogram, evaluation of intravenous access, prostatic massage and expressed prostatic fluid culture, workup for immunodeficiency, consultation with microbiologist	Keywords: *recurrent bacteremia*, *ESBL E coli*; DDx: chronic granulomatous disease, gastroenteritis, coagulase-negative staphylococcus, amebiasis, fluke infection	Dx: deep-seated infection; DDx: recurrent urinary tract infection; workup suggested: whole-body gallium scan and/or ^18^FDG-PET scan (no other specialist consulted)
Patient 5	Metronidazole-related encephalopathy (MRI of the brain showed hyperintensities at the dentate nuclei)	Dx: seizure; DDx: need to watch electrolyte disturbances, medications, infection, stroke, intracranial mass and/or lesion, Lewy body dementia; workup suggested: EEG, MRI of the brain, lumbar puncture, review of medications	Dx: seizure; DDx: need to watch electrolyte disturbances, medications, infection, stroke, intracranial mass and/or lesion, Lewy body dementia; workup suggested: EEG, MRI of the brain, lumbar puncture, review of medications	Dx: metronidazole-induced encephalopathy; DDx: stroke, electrolyte disturbances, intracranial infection, subdural hematoma and seizures related to Lewy body dementia; workup suggested: discontinuation of metronidazole, lumbar puncture, MRI of the brain, EEG, consultation with neurologist	Keywords: *seizure*, *dementia*, *metronidazole*; DDx: cerebral amyloid angiopathy, human prion diseases, Alzheimer disease, vascular dementia, measles	Dx: Lewy body dementia–related seizures; DDx: brain neoplasm; workup suggested: MRI of the brain (neurologist had been consulted to arrange EEG)
Patient 6	Gastric diffuse large B cell lymphoma (gastric biopsy confirmation)	Dx: malignant neoplasm; DDx: gastroparesis and/or functional dyspepsia, postviral syndrome (COVID-19), chronic kidney disease, age-related physiological changes, medication adverse effects; workup suggested: CT or MRI of the abdomen and pelvis, EGD or colonoscopy, biopsy of retroperitoneal lymph node, consult dietician	Dx: malignant neoplasm or inflammatory and/or autoimmune condition; DDx: gastroparesis and/or functional dyspepsia, postviral syndrome (COVID-19), chronic kidney disease, age-related physiological changes, medication adverse effects; workup suggested: CT or MRI abdomen and pelvis, EGD or colonoscopy, biopsy of retroperitoneal lymph node, consult dietician	Dx: malignant neoplasm (eg, lymphoma); DDx: gastroparesis, chronic infection and/or autoimmune disease, anemia of chronic illness; workup suggested: EGD and/or colonoscopy, CT and/or MRI of the abdomen, lymph node biopsy, consultation with hematologist and/or clinical oncologist	Keywords: *anorexia*, *lymphadenopathy*; DDx: *Bartonella* infection, non-Hodgkin lymphoma, infectious mononucleosis, Hodgkin disease, adult Still disease	Dx: polymyalgia rheumatica; DDx: malignant neoplasia, (eg, lymphoma); workup suggested: whole body ^18^FDG-PET (rheumatologist had been consulted, suggested careful workup for the lymphadenopathy with ^18^FDG-PET)

Abbreviations: ANA, antinuclear antibody; ANCA, antineutrophil cytoplasmic antibody; BME, bone marrow examination; CHF, congestive heart failure; CT, computed tomography; Dx, diagnosis; DDx, differential diagnosis; *E coli*, *Eschericha coli*; EGD, esophagogastroduodenoscopy; EEG, electroencephalogram; ESBL, extended-spectrum β-lactamase; ^18^FDG, fluorodeoxyglucose; MRI, magnetic resonance imaging; PET, positron emission tomography; UTI, urinary tract infection.

## Discussion

Overall, GPT-4 has potential clinical use in older patients without a definitive clinical diagnosis after 1 month but requires comprehensive entry of demographic and clinical (including radiological and pharmacological) information. GPT-4 may increase confidence in diagnosis and earlier commencement of appropriate treatment, alert clinicians missing important diagnoses, and offer suggestions similar to specialists to achieve the correct clinical diagnosis, which has potential value in low-income countries with lack of specialist care. Clinicians need to be aware that GPT-4 is limited in multifocal infection, and the suggested management plan should be correlated with clinical context, as suggestions may be redundant. Clinicians should consider a drug review and review the possible diagnosis of malignant disease if suggested.

This study has several limitations. First, GPT-4 may not detect 2 focuses of infection or pinpoint the source of recurrent infection. Second, GPT-4 did not suggest the use of gallium scan or 18-fluorodeoxyglucose positron emission tomography to look for infections or malignant neoplasms in all but 1 patient. Third, some investigations may not be appropriate (eg, temporal artery biopsy in the absence of typical symptoms of giant cell arteritis). Overall, our findings suggest that the use of AI in diagnosis is both promising and challenging.

## Notes

Supplement 1. **eMethods.** Patient Histories Entered and GPT-4 Reply

Supplement 2.
Data Sharing Statement
