# Supplementary material for: Use of GPT-4 to Analyze Medical Records of Patients With Extensive Investigations and Delayed Diagnosis
Source: JAMA Netw Open. 2023 Aug 14;6(8):e2325000. doi: 10.1001/jamanetworkopen.2023.25000 (PMC10425828; doi:10.1001/jamanetworkopen.2023.25000)
Supplement: Supplement 1. — eMethods. Patient Histories Entered and GPT-4 Reply [file jamanetwopen-e2325000-s001.pdf]

Supplementary Online Content

Shea YF, Lee CMY, Ip WCT, Luk DWA, Wong SSW. Use of GPT-4 to analyze medical records of patients with extensive investigations and delayed diagnosis. *JAMA Netw Open*. 2023;6(8):e2325000. doi:10.1001/jamanetworkopen.2023.25000

eMethods. Patient Histories Entered and GPT-4 Reply

This supplementary material has been provided by the authors to give readers additional information about their work.

© 2023 Shea YF et al. *JAMA Network Open*.

eMethods. Patient Histories Entered and GPT-4 Reply

Patient 1

Upon presentation:

For the following medical history, we would like to ask you some questions at the end.

An 83-year-old woman was admitted for fever, dysuria and right loin pain.

She presented with one day history of fever, dysuria and right loin pain. There was no shortness of breath, productive cough or diarrhoea. Physical examination showed blood pressure of 157/86 mmHg, pulse was 86 per minute with only right loin pain on percussion. Chest-X-ray showed no consolidation. Kidney-ureter-bladder x-ray only showed left hip implant without any urinary stone. Electrocardiogram was unremarkable. White cell count was 9.96 $x10^9$/L. Urine routine microscopy showed elevated white cell count. Blood culture was normal. Patient was prescribed with augmentin.

She had a past medical history of osteoporosis, gastrointestinal stromal tumor at the gastric fundus, history of fracture hips with screw fixation and proximal femoral nailing, seronegative rheumatoid arthritis or polymyalgia rheumatica on long-term prednisolone tablet 5 mg daily and history of laparoscopic cholecystectomy. She had no known drug allergy. She lived with her son and grandson before admission. She walked with a stick.

With the above history,

1. What is your primary diagnosis?
2. What are your differential diagnoses?
3. Any further work-up that you would suggest?

GPT-4's reply:

I'm not a doctor, but based on the information provided, I can offer some suggestions for your consideration. Please consult with a healthcare professional for a definitive diagnosis and personalized advice.

1. Primary diagnosis:

The primary diagnosis could be a urinary tract infection (UTI), considering her symptoms of fever, dysuria (painful urination), and right loin pain, along with the elevated white cell count in her urine.

2. Differential diagnoses:

- Pyelonephritis: Infection of the kidney, which may present with similar symptoms to a UTI and can be a complication of an untreated or severe UTI.

- Urolithiasis: Urinary stones causing urinary obstruction and inflammation. However, the kidney-ureter-bladder x-ray did not show any urinary stones.

- Musculoskeletal pain: Given her history of osteoporosis, fractures, and rheumatoid arthritis or polymyalgia rheumatica, it's possible that her right loin pain could be related to a musculoskeletal issue.

3. Further work-up suggestions:

© 2023 Shea YF et al. *JAMA Network Open*.

- Urine culture and sensitivity: To identify the causative organism and appropriate antibiotic treatment.

- Renal ultrasound: To assess for any signs of kidney infection, hydronephrosis, or other abnormalities that may not have been visible on the kidney-ureter-bladder x-ray.

- Complete blood count (CBC) and C-reactive protein (CRP): To evaluate the extent of inflammation/infection.

- Review of her medications and medical history: To ensure there are no medication interactions or other factors that could be contributing to her symptoms.

Again, I'm not a doctor, but these suggestions may be helpful for you to discuss further with a healthcare professional.

One week after admission:

For the following medical history, we would like to ask you some questions at the end.

An 83-year-old woman was admitted for fever, dysuria and right loin pain.

She presented with one day history of fever, dysuria and right loin pain. There was no shortness of breath, productive cough or diarrhoea. Physical examination showed blood pressure of 157/86 mmHg, pulse was 86 per minute with only right loin pain on percussion. Chest-X-ray showed no consolidation. Kidney-ureter-bladder x-ray only showed left hip implant without any urinary stone. Electrocardiogram was unremarkable. White cell count was 9.96 $x10^9$/L. Urine routine microscopy showed elevated white cell count. Blood culture was normal. Patient was prescribed with augmentin.

The patient remained febrile despite 2 days of augmentin and 5 days of tazocin. Urine culture yielded *Enterococcus faecium*. Vancomycin was added. C-reactive protein was elevated to 11.23 mg/dL. Additional spine X-ray was performed but no lytic lesion was noted.

She had a past medical history of osteoporosis, gastrointestinal stromal tumor at the gastric fundus, history of fracture hips with screw fixation and proximal femoral nailing, seronegative rheumatoid arthritis or polymyalgia rheumatica on long-term prednisolone tablet 5 mg daily and history of laparoscopic cholecystectomy. She had no known drug allergy. She lived with her son and grandson before admission. She walked with a stick.

With the above history,

1. What is your primary diagnosis?
2. What are your differential diagnoses?
3. Any further work-up that you would suggest?

GPT-4's reply:

I'm not a doctor, but based on the information provided, I can offer some suggestions for your consideration. Please consult with a healthcare professional for a definitive diagnosis and personalized advice.

1. Primary diagnosis:

© 2023 Shea YF et al. *JAMA Network Open*.

Considering the patient's persistent symptoms and the positive urine culture for Enterococcus faecium, the primary diagnosis could be a complicated urinary tract infection (UTI) or pyelonephritis that is not responding to the initial antibiotic treatments.

2. Differential diagnoses:

- Bacteremia or sepsis: The patient's elevated C-reactive protein and persistent fever despite antibiotics may suggest the presence of systemic infection.
- Intra-abdominal infection: Given her history of laparoscopic cholecystectomy, it is essential to rule out any intra-abdominal infection that could be causing her persistent fever.
- Musculoskeletal infection: Since the patient has a history of fracture hips with screw fixation and proximal femoral nailing, it's important to consider the possibility of an infection in the hardware or surrounding tissue.

3. Further work-up suggestions:

- Repeat blood culture: To check for any signs of bacteremia or sepsis.
- Imaging studies: Consider a CT scan of the abdomen and pelvis to rule out any intra-abdominal infection or other abnormalities. Additionally, a bone scan or MRI might be helpful to evaluate for any musculoskeletal infection around the hip implant or other areas.
- Consult an infectious disease specialist: For guidance on the appropriate management of the Enterococcus faecium UTI and any potential complications.
- Reassess antibiotic therapy: If the patient's condition does not improve, consider adjusting the antibiotic regimen based on the results of antibiotic sensitivity testing.

Again, I'm not a doctor, but these suggestions may be helpful for you to discuss further with a healthcare professional.

Before final diagnosis:

For the following medical history, we would like to ask you some questions at the end.

An 83-year-old woman was admitted for pyrexia of unknown origin. She developed fever whenever the antibiotic was stopped.

She was initially admitted for fever and right loin pain. The patient remained febrile despite 2 days of augmentin, 5 days of tazocin and 17 days of meropenem. Urine culture yielded *Enterococcus faecium*. She was treated as pyelonephritis. Vancomycin was added but fever persisted. Computed tomography of the abdomen was performed and there was no evidence of liver abscess, psoas abscess or hydronephrosis. There was an incidental finding of a 3.9 cm abdominal aortic aneurysm at the level of crus of diaphragm. Physical examination was unremarkable. CRP was elevated to 14.44 mg/dL. Total white cell count was 12.67 x 10^9/L

She had a past medical history of osteoporosis, gastrointestinal stromal tumor at the gastric fundus, history of fracture hips with screw fixation and proximal femoral nailing, seronegative rheumatoid arthritis or polymyalgia rheumatica on long-term prednisolone tablet 5 mg daily and history of laparoscopic cholecystectomy. She had no known drug allergy. She lived with her son and grandson before admission. She walked with a stick.

With the above history,

1. What is your primary diagnosis?

© 2023 Shea YF et al. *JAMA Network Open*.

2. What are your differential diagnoses?
3. Any further work-up that you would suggest?

GPT-4's reply:

1. **Primary diagnosis**: Based on the patient's history and presentation, the primary diagnosis is a persistent or complicated urinary tract infection (UTI) or pyelonephritis caused by Enterococcus faecium. The patient has a history of fever and right loin pain, and her urine culture reveals Enterococcus faecium. Although she has been treated with various antibiotics, her fever has persisted.

2. **Differential diagnoses**:
   - Intra-abdominal abscess: Although the CT scan did not show evidence of liver abscess, psoas abscess, or hydronephrosis, it is still possible that there is an undetected intra-abdominal source of infection.
   - Endocarditis: Enterococcus faecium can be a cause of infective endocarditis, and the patient's persistent fever and elevated inflammatory markers could be suggestive of this diagnosis.
   - Vascular infection: The incidental finding of an abdominal aortic aneurysm raises the possibility of an infected aneurysm or another vascular infection.
   - Osteomyelitis: Given the patient's history of fracture hips with screw fixation and proximal femoral nail, as well as her chronic use of prednisolone, she may be at risk of developing osteomyelitis.
   - Drug fever: While less likely, it is important to consider that the patient's fever could be due to drug fever, which could be related to her antibiotic treatments.

3. **Further work-up**:
   - Blood cultures: To assess for bacteremia and the possibility of endocarditis or vascular infection.
   - Transthoracic or transesophageal echocardiogram: To evaluate for vegetations associated with infective endocarditis.
   - Repeat imaging of the abdomen and pelvis: Consider a repeat CT scan or MRI to better assess for any undetected intra-abdominal source of infection or complications related to the aneurysm.
   - Imaging of the hip and femur: To evaluate for possible osteomyelitis at the site of previous fractures and hardware.
   - Antibiotic susceptibility testing: To ensure that the patient is receiving appropriate antibiotic therapy for the Enterococcus faecium infection.
   - Consultation with an infectious disease specialist: To help guide the management of this complex case and determine the most appropriate course of action.

Clinical outcome:

Gallium scan, performed 16 days after admission, subsequently showed an interval dilatation of descending aorta (5 cm) at diaphragmatic crus with patchy uptake at the right side and anterior aspect of aorta. Increased uptake was also noted at bilateral kidneys with right side more than left side reflecting resolving right acute pyelonephritis with superimposed drug induced bilateral nephritis. CT aortogram was performed and showed a static ectatic distal

© 2023 Shea YF et al. *JAMA Network Open*.

descending aorta up to 4cm at diaphragmatic crus level. The rest of the included aorta had normal caliber.

Blood culture taken during breakthrough fever later grew extended-spectrum beta-lactamase producing *Salmonella enteritidis*. Subsequent whole body positron emission tomography (PET) noted hypermetabolic periaortic activity (SUVmax = 12) surrounding lower thoracic aorta through the diaphragmatic hiatus with progression of cystic component anteriorly compatible with mycotic aneurysm (depth 2.8 cm). The aneurysmal sac measured a maximal diameter of 5.9 cm perpendicular to the long axis of aorta. A diagnosis of ESBL producing *Salmonella enteritidis*-related mycotic aneurysm was made. Endovascular aneursymal repair (EVAR) was performed 44 days after the initial admission. The patient was put on intravenous ertapenem with oral azithromycin for 4 weeks, then switched to life-long oral azithromycin as maintenance therapy. Repeated PET showed reduced SUV max and the size of the mycotic aneurysm reduced to 2.4 cm.

© 2023 Shea YF et al. *JAMA Network Open*.

Patient 2
Upon presentation

For the following medical history, we would like to ask you some questions at the end.

An 84-year-old woman was admitted due to a three-week history of generalized weakness.

The patient had a weight loss of 5 kg in one month and bilateral knee pain. Physical examination showed body temperature of 37.7 degrees and left knee arthritis. Investigations showed hemoglobin of 9.7 g/dL, white cell count of 13 x 10^9/L, platelet of 621 x 10^9/L. C-reactive protein was 7.56 mg/L. Erythrocyte sedimentation rate was 128 mm/hr. Thyroid function test was normal. X-ray of the knee showed osteoarthritis. Urine routine microscopy was normal. Chest X-ray was normal.

She had a past medical history of hypertension, diabetes mellitus and hyperlipidemia. She had no known drug allergy. She walked with a frame and lived with her son.

With the above history,

1. What is your primary diagnosis?
2. What are your differential diagnoses?
3. Any further work-up that you would suggest?

GPT-4's reply:
I'm not a doctor, but based on the information provided, I can offer some suggestions for your consideration. Please consult with a healthcare professional for a definitive diagnosis and personalized advice.

1. Primary diagnosis:
Given the generalized weakness, weight loss, elevated inflammatory markers, and anemia, the primary diagnosis could be an underlying infection or inflammatory condition.

2. Differential diagnoses:

- Inflammatory arthritis: The patient's elevated erythrocyte sedimentation rate (ESR), C-reactive protein (CRP), and left knee arthritis may indicate an inflammatory arthritis, such as rheumatoid arthritis or polymyalgia rheumatica.
- Infection: The elevated white cell count and inflammatory markers may suggest an underlying infection, although no specific source has been identified yet.
- Malignancy: Unexplained weight loss and anemia could be indicative of an underlying malignancy. In this age group, a thorough evaluation is needed to rule out malignancy as a cause of her symptoms.
- Anemia of chronic disease: The patient's anemia could be related to a chronic inflammatory condition or malignancy, which may contribute to her generalized weakness and weight loss.

3. Further work-up suggestions:

- Additional blood tests: Comprehensive metabolic panel, iron studies, vitamin B12, and folate levels to assess the cause of anemia.
- Rheumatologic evaluation: Autoimmune markers, such as antinuclear antibodies (ANA) and rheumatoid factor (RF), to test for the presence of an autoimmune or inflammatory condition.

© 2023 Shea YF et al. *JAMA Network Open*.

- Imaging studies: Consider a CT scan of the chest, abdomen, and pelvis to rule out any occult malignancy or hidden infection.
- Bone marrow biopsy: If the cause of anemia and elevated platelet count remains unexplained after the initial work-up, a bone marrow biopsy may be warranted to assess for any hematologic disorder or malignancy.
- Infectious disease consultation: If there is a suspicion of an underlying infection, consultation with an infectious disease specialist could be valuable.

Again, I'm not a doctor, but these suggestions may be helpful for you to discuss further with a healthcare professional.

One week after presentation:
For the following medical history, we would like to ask you some questions at the end.

An 84-year-old woman was admitted due to a three-week history of generalized weakness.

The patient had a weight loss of 5 kg in one month and bilateral knee pain. Physical examination showed body temperature of 37.7 degrees and left knee arthritis. Investigations showed hemoglobin of 9.7 g/dL, white cell count of 13 x 10^9/L, platelet of 621 x 10^9/L. C-reactive protein was 7.56 mg/L. Erythrocyte sedimentation rate was 128 mm/hr. Thyroid function test was normal. X-ray of the knee showed osteoarthritis. Urine routine microscopy was normal. Chest X-ray was normal.

Left knee arthrocentesis was performed. Joint fluid yielded a white cell count of 17090 x 10^6/L and cytology showed calcium pyrophosphate crystals. The patient was managed as pseudogout. She was given colchicine and paracetamol. Her knee pain improved.

However, she continued to experience generalized weakness. A repeat of the physical examination showed no evidence of lymphadenopathy, abdominal mass or breast mass. Iron profile was checked and showed anemia of chronic illness and there was folic acid deficiency. Further work-up included autoimmune markers: antinuclear antibody, anti-neutrophil cytoplasmic antibody, rheumatoid factor and anti-cyclic citrullinated peptide antibody, all of which were negative. Tumor markers, including carcinoembryonic antigen, alpha fetoprotein, Ca19-9, Ca 15.3 and Ca 125, were normal.

She had a past medical history of hypertension, diabetes mellitus and hyperlipidemia. She had no known drug allergy. She walked with a frame and lived with her son.

With the above history,
1. What is your primary diagnosis?
2. What are your differential diagnoses?
3. Any further work-up that you would suggest?

GPT-4's reply:
I'm not a doctor, but based on the information provided, I can offer some suggestions for your consideration. Please consult with a healthcare professional for a definitive diagnosis and personalized advice.

1. Primary diagnosis:

© 2023 Shea YF et al. *JAMA Network Open*.

With the additional information provided, the patient's knee pain has been addressed as pseudogout, but the primary diagnosis for her generalized weakness remains unclear. It could be related to the anemia of chronic illness and folic acid deficiency.

2. Differential diagnoses:

- Chronic fatigue syndrome: The patient's persistent generalized weakness may be due to chronic fatigue syndrome, which is a complex disorder characterized by extreme fatigue that doesn't improve with rest and may worsen with physical or mental activity.
- Unexplained anemia: The anemia of chronic illness and folic acid deficiency may be contributing factors to her generalized weakness, but the underlying cause remains unclear.
- Malnutrition or vitamin deficiency: The patient's weight loss and folic acid deficiency may suggest underlying malnutrition or other vitamin deficiencies that could contribute to her symptoms.
- Age-related physiological changes: The patient's generalized weakness could be related to normal age-related changes in muscle mass, energy levels, and overall health.

3. Further work-up suggestions:

- Nutritional assessment: A thorough nutritional assessment to identify any deficiencies or malnutrition that may contribute to her symptoms. This could include checking vitamin D and B12 levels, as well as other relevant micronutrients.
- Gastrointestinal evaluation: An endoscopic evaluation may be warranted to rule out any occult gastrointestinal bleeding or malabsorption that could contribute to her anemia and weight loss.
- Sleep study: If the patient reports poor sleep quality or daytime sleepiness, a sleep study could help rule out sleep disorders, such as sleep apnea, which could contribute to her generalized weakness.
- Geriatric assessment: A comprehensive geriatric assessment to evaluate her overall functional status, cognitive function, and other factors that could contribute to her symptoms and help guide management strategies.

Again, I'm not a doctor, but these suggestions may be helpful for you to discuss further with a healthcare professional.

Before the final diagnosis:

For the following medical history, we would like to ask you some questions at the end.

An 84-year-old woman was admitted due to a three-week history of generalized weakness.
She had a past medical history of hypertension, diabetes mellitus and hyperlipidemia. She had no known drug allergy. She walked with a frame and lived with her son. The patient had a weight loss of 5 kg in one month and bilateral knee pain. Physical examination showed left knee arthritis. Investigations showed hemoglobin of 9.7 g/dL, white cell count of 13 x 10^9/L, platelet of 621 x 10^9/L. C-reactive protein was 7.56 mg/L. Erythrocyte sedimentation rate was 128 mm/hr. X-ray of the knee showed osteoarthritis. Left knee arthrocentesis was performed. Joint fluid yielded a white cell count of 17090 x 10^6/L and cytology showed calcium pyrophosphate crystals. The patient was managed as pseudogout. She was given colchicine and paracetamol. Her knee pain improved.

© 2023 Shea YF et al. *JAMA Network Open*.

However, she continued to experience generalized weakness. In the view of weight loss, whole body positron emission-computed tomography was performed, showing 1) an incidental finding of a moderately hypermetabolic nodule in the deep lobe of the left parotid gland, which could represent a primary parotid lesion or a lymph node; 2) non-specific ground-glass nodule in the left upper lobe; 3) no hypermetabolic malignancy but given the intense colonic muscular activity, the physiologic uptake may obscure mucosal uptake abnormality. Further work-up included autoimmune markers: antinuclear antibody, anti-neutrophil cytoplasmic antibody, rheumatoid factor and anti-cyclic citrullinated peptide antibody, all of which were negative. Red blood cell folate deficiency was found and folic acid was prescribed.

The geriatric team was consulted. Review of history noted bilateral shoulder and hip stiffness. There was no jaw claudication or bitemporal headache or impaired visual acuity.

With the above history,

1. What is your primary diagnosis?
2. What are your differential diagnoses?
3. Any further work-up that you would suggest?

GPT-4's reply:

1. Primary Diagnosis:

Based on the patient's age, history, clinical presentation, and laboratory findings, the primary diagnosis is polymyalgia rheumatica (PMR). PMR is an inflammatory condition that typically affects older individuals and is characterized by muscle pain and stiffness, particularly around the shoulders and hips. The patient's elevated erythrocyte sedimentation rate (ESR) and C-reactive protein (CRP) levels, as well as her symptoms of bilateral shoulder and hip stiffness, support this diagnosis.

2. Differential Diagnoses:

- Pseudogout: The patient's left knee arthritis and the presence of calcium pyrophosphate crystals in the joint fluid suggest pseudogout. However, pseudogout typically does not cause bilateral shoulder and hip stiffness.
- Paraneoplastic syndrome: The patient's weight loss and the findings on the positron emission-computed tomography (PET-CT) scan, such as the hypermetabolic nodule in the left parotid gland and the ground-glass nodule in the left upper lobe, raise the possibility of an underlying malignancy. Paraneoplastic syndromes are a group of symptoms that occur as a result of substances produced by the tumor or the body's immune response to the tumor, and they can also cause musculoskeletal symptoms.
- Inflammatory arthritides: Conditions like rheumatoid arthritis or seronegative spondyloarthropathies could be considered, but the patient's negative autoimmune markers make these diagnoses less likely.

3. Further Work-up:

- Temporal artery biopsy: Although the patient has no signs of jaw claudication, bitemporal headache, or impaired visual acuity, her age and symptoms still raise the possibility of giant cell arteritis (GCA), which can be associated with PMR. A temporal artery biopsy can help rule out or confirm GCA.

© 2023 Shea YF et al. *JAMA Network Open*.

- Further evaluation of the PET-CT findings: The patient should undergo further diagnostic work-up for the hypermetabolic nodule in the left parotid gland and the ground-glass nodule in the left upper lobe. This may include a fine-needle aspiration biopsy of the parotid lesion and a follow-up chest CT scan with or without biopsy of the lung lesion.
- Trial of low-dose corticosteroids: If PMR is strongly suspected, a trial of low-dose corticosteroids (such as prednisone) can be initiated. A rapid and significant improvement in symptoms after corticosteroid initiation is supportive of a PMR diagnosis.

Clinical outcome:
Weakness improved with prednisolone 20 mg daily orally for 2 months and then it was gradually tapered down to 2.5 mg daily orally. The patient was diagnosed with polymyalgia rheumatica.

© 2023 Shea YF et al. *JAMA Network Open*.

Patient 3

Upon presentation:

For the following medical history, we would like to ask you some questions at the end.

An 86-year-old man was admitted with lower limb weakness and deranged liver function.

He was brought by his son to the accident and emergency department because of lower limb weakness and borderline blood pressure (104/60 mmHg). Physical examination showed the following: blood pressure of 149/88 mmHg, body temperature of 36.2 degrees, oxygen saturation of 96% on 2 liters of nasal cannula. Respiratory examination revealed expiratory wheeze. Abdominal, cardiovascular and neurological examinations were unremarkable. He had a past medical history of hypertension, benign prostatic hyperplasia, transient ischemic attack, and Alzheimer's disease. He lived with his son. He was a non-drinker and ex-smoker.

After admission, the patient was noted to have a deranged renal function test with creatinine of 143 umol/L, mixed pattern of liver function derangement (total bilirubin: 104 umol/L, alkaline phosphatase: 236 U/L, alanine aminotransferase: 93 U/L, aspartate aminotransferase: 210 U/L, and gamma glutamyltransferase: 347 U/L). Complete blood picture was normal. Blood culture was negative. Chest X-ray showed cardiomegaly with bilateral reticulations.

With the above history,

1. What is your primary diagnosis?
2. What are your differential diagnoses?
3. Any further work-up that you would suggest?

GPT-4's reply:

I'm not a doctor, but based on the information provided, I can offer some suggestions for your consideration. Please consult with a healthcare professional for a definitive diagnosis and personalized advice.

1. Primary diagnosis:

The primary diagnosis is not clear from the information provided, but the patient presents with lower limb weakness, deranged liver function, and renal dysfunction. These symptoms may be related to multiple underlying conditions.

2. Differential diagnoses:

- Congestive heart failure: The cardiomegaly and bilateral reticulations on chest X-ray could suggest congestive heart failure, which may cause lower limb weakness due to poor perfusion and fluid retention.
- Liver disease: The mixed pattern of liver function derangement may indicate an underlying liver disease, which could be related to viral hepatitis, medications, or other causes.
- Kidney disease: The elevated creatinine level suggests impaired renal function, which could be related to chronic kidney disease, acute kidney injury, or another renal disorder.
- Autoimmune or connective tissue disease: The patient's symptoms could be related to an underlying autoimmune or connective tissue disease, such as lupus or vasculitis, which may cause multiorgan involvement.

© 2023 Shea YF et al. *JAMA Network Open*.

3. Further work-up suggestions:

- Echocardiogram: To assess cardiac function and evaluate for signs of congestive heart failure.
- Abdominal ultrasound: To evaluate the liver, gallbladder, and kidneys for any structural abnormalities or signs of disease.
- Liver function evaluation: Viral hepatitis serologies, autoimmune liver markers, and a review of the patient's medications for potential hepatotoxicity.
- Renal function evaluation: Urinalysis, urine protein-to-creatinine ratio, and renal ultrasound to further assess kidney function and potential causes of renal impairment.
- Autoimmune/connective tissue disease evaluation: Additional blood tests, such as antinuclear antibody (ANA), antineutrophil cytoplasmic antibodies (ANCA), and complement levels, to assess for the presence of an autoimmune or connective tissue disease.

Again, I'm not a doctor, but these suggestions may be helpful for you to discuss further with a healthcare professional.

One week after presentation:

For the following medical history, we would like to ask you some questions at the end.

An 86-year-old man was admitted with lower limb weakness, deranged liver function and thrombocytopenia.

He was brought by his son to the accident and emergency department because of lower limb weakness and borderline blood pressure (104/60 mmHg). Physical examination showed the following: blood pressure of 149/88 mmHg, body temperature of 36.2 degrees, oxygen saturation of 96% on 2 liters of nasal cannula. Respiratory examination revealed expiratory wheeze. Abdominal, cardiovascular and neurological examinations were unremarkable. He had a past medical history of hypertension, benign prostatic hyperplasia, transient ischemic attack, and Alzheimer’s disease. He lived with his son. He was a non-drinker and ex-smoker.

After admission, the patient was noted to have a deranged renal function test with creatinine of 143 umol/L, mixed pattern of liver function derangement (total bilirubin: 104 umol/L, alkaline phosphatase: 236 U/L, alanine aminotransferase: 93 U/L, aspartate aminotransferase: 210 U/L, and gamma glutamyltransferase: 347 U/L). Complete blood picture was normal. Blood culture was negative. Chest X-ray showed cardiomegaly with bilateral reticulations.

The platelet count gradually declined to 32 x 10^9/L. Hepatitis markers for hepatitis A, B, C and E were negative. Ultrasound of the liver was unremarkable. Computed tomography of the abdomen showed splenomegaly but no evidence of liver abscess, no periportal edema. There was a thickened gallbladder wall up to 10 mm (which could be related to hypoalbuminemia). Tumor markers only showed slightly elevated carcinoembryonic antigen 9.8 ng/ml but negative results for alpha-fetoprotein and Ca19-9. EBV PCR was less than 100 IU/ml and CMV & adenovirus PCR was negative. Serology for Brucella, Burkholderia pseudomallei Ab IgM, leptospira and melioidosis were negative. Blood for cryptococcal antigen and aspergillus antigen were negative. HIV serology was negative. Anti-nuclear antibody was positive at 1/160 but anti-smooth muscle antibody, anti-mitochondrial antibody, anti-neutrophil cytoplasmic antibody, C3 and C4 were unremarkable. One sample of urine and One sample of early morning gastric aspirate for acid fast bacilli smear were negative.

© 2023 Shea YF et al. *JAMA Network Open*.

With the above history,

1. What is your primary diagnosis?
2. What are your differential diagnoses?
3. Any further work-up that you would suggest?

GPT-4's reply:

I'm not a doctor, but based on the information provided, I can offer some suggestions for your consideration. Please consult with a healthcare professional for a definitive diagnosis and personalized advice.

1. Primary diagnosis:

The primary diagnosis is not clear from the information provided. The patient presents with lower limb weakness, deranged liver function, renal dysfunction, and thrombocytopenia. The extensive work-up conducted has ruled out many potential causes, making it more challenging to pinpoint a specific diagnosis.

2. Differential diagnoses:

- Hypoalbuminemia-related complications: The thickened gallbladder wall may be related to hypoalbuminemia, which could also contribute to the deranged liver function and renal dysfunction.
- Autoimmune or connective tissue disease: The positive ANA test may indicate an underlying autoimmune or connective tissue disease that could cause multiorgan involvement, even though other specific autoantibodies are unremarkable.
- Myelodysplastic syndrome or another bone marrow disorder: The patient's thrombocytopenia and splenomegaly could be related to a bone marrow disorder, which may also cause deranged liver function and renal dysfunction.
- Paraneoplastic syndrome: The slightly elevated carcinoembryonic antigen (CEA) may indicate an occult malignancy, which could cause a paraneoplastic syndrome affecting multiple organ systems.

3. Further work-up suggestions:

- Bone marrow biopsy: To assess for myelodysplastic syndrome or another bone marrow disorder as the cause of the patient's thrombocytopenia and splenomegaly.
- Imaging studies: Additional imaging, such as a CT scan of the chest and pelvis, to evaluate for occult malignancies that may be causing a paraneoplastic syndrome.
- Serum protein electrophoresis and immunofixation: To evaluate for the presence of monoclonal proteins that could be associated with plasma cell dyscrasias or other hematologic malignancies.
- Rheumatology consultation: To further evaluate the patient's positive ANA test and consider additional autoimmune or connective tissue disease testing.

Again, I'm not a doctor, but these suggestions may be helpful for you to discuss further with a healthcare professional.

Before final diagnosis:

For the following medical history, we would like to ask you some questions at the end.

© 2023 Shea YF et al. *JAMA Network Open*.

An 86-year-old man was admitted with lower limb weakness, deranged liver function and thrombocytopenia.

He was brought by his son to the accident and emergency department because of lower limb weakness and borderline blood pressure (104/60 mmHg). Physical examination showed the following: blood pressure of 149/88 mmHg, body temperature of 36.2 degrees, oxygen saturation of 96% on 2 liters of nasal cannula. Respiratory examination revealed expiratory wheeze. Abdominal, cardiovascular and neurological examinations were unremarkable. Chest X-ray showed cardiomegaly with bilateral reticulations. He had a past medical history of hypertension, benign prostatic hyperplasia, transient ischemic attack, and Alzheimer's disease. He lived with his son. He was a non-drinker and ex-smoker.

After admission, the patient was noted to have a deranged renal function test with creatinine of 143 umol/L, mixed pattern of liver function derangement (total bilirubin: 104 umol/L, alkaline phosphatase: 236 U/L, alanine aminotransferase: 93 U/L, aspartate aminotransferase: 210 U/L, and gamma glutamyltransferase: 347 U/L). Ultrasound of the kidney was unremarkable and there was no hydronephrosis. Ultrasound of the liver was unremarkable. Computed tomography of the abdomen showed splenomegaly but no evidence of liver abscess, no periportal edema. There was a thickened gallbladder wall up to 10 mm (which could be related to hypoalbuminemia).

Over his stay, the patient had persistent thrombocytopenia ranging from 42-68 x 10^9/L. The clotting profile was normal. Fibrinogen was 1.32 g/L. He was found to have normochromic and normocytic anemia of 9.1 g/dL. Iron profile showed anemia of chronic illness with ferritin of 4427 pmol/L. Fasting triglycerides was 3.4 mmol/L. Four sets of early morning gastric aspirates were negative for acid-fast bacilli. Four sets of early morning urine were negative for acid-fast bacilli smear, but one sample showed positive polymerase chain reaction (PCR) for *Mycobacterium tuberculosis*. One blood culture for acid-fast bacilli was smear negative. Interferon Gamma release assay was negative. Other extensive microbiological work-up was negative including the following: Leptospira IgM, Hantaan virus Ab, Q fever serology, Typhus antibody, adenovirus PCR, acute hepatitis serology including (Hepatitis A and E) IgM, and negative Hepatitis B PCR. The patient was given intravenous meropenem and metronidazole. He was also given a course of oral doxycycline to cover suspected atypical infection.

There was a diagnostic dilemma of whether to treat possible disseminated tuberculosis as the patient had liver function derangement and would be risky for anti-tuberculous treatment. The isolated positive urine PCR for tuberculosis could be false positive. So the decision was to arrange whole-body positron emission tomography or gallium scan for any deep-seated infection. Bone marrow examination was performed but the sample was inadequate for assessment. Result of trephine biopsy assessment was pending. Peripheral blood smear showed leukoerythroblastic blood picture, mild anisopoikilocytosis, occasional elliptocytes and targets cells, occasional myelocytes and atypical lymphocytes, and rare nucleated red blood cells.

With the above history,

4. What is your primary diagnosis?
5. What are your differential diagnoses?
6. Any further work-up that you would suggest?

GPT-4's reply:

1. **Primary Diagnosis:** Given the patient's history and laboratory findings, a primary diagnosis is challenging to determine without additional information. However, one

© 2023 Shea YF et al. *JAMA Network Open*.

possibility to consider could be disseminated tuberculosis, given the positive PCR for Mycobacterium Tuberculosis in the urine sample. The patient's age and comorbidities may make him more susceptible to infections, and tuberculosis can present with a variety of symptoms.

2. **Differential Diagnoses:** There are several differential diagnoses to consider based on the patient's presentation:

   - Autoimmune or inflammatory conditions (e.g., vasculitis or connective tissue diseases) that can cause a deranged liver function, thrombocytopenia, and anemia.
   - Hematologic malignancies such as lymphoma or leukemia, which could explain the leukoerythroblastic blood picture and thrombocytopenia.
   - Liver disease or cirrhosis, which could contribute to deranged liver function, hypoalbuminemia, and thrombocytopenia.
   - Infections other than tuberculosis, such as atypical bacterial, viral or fungal infections, which could cause a variety of systemic symptoms and deranged laboratory values.
   - Paraneoplastic syndromes related to an undiagnosed malignancy, which can cause a variety of nonspecific symptoms and laboratory abnormalities.

   0. Further Work-up: Additional tests and procedures to consider might include:

   - Whole-body positron emission tomography (PET) or gallium scan, as mentioned in the history, to identify any deep-seated infection or potential malignancy.
   - Repeat bone marrow biopsy to obtain an adequate sample for assessment, which could help provide more information about potential hematologic malignancies or other causes of the patient's symptoms.
   - Serological tests for autoimmune or inflammatory conditions, such as antinuclear antibodies (ANA), rheumatoid factor (RF), and anti-neutrophil cytoplasmic antibodies (ANCA).
   - Additional imaging, such as a chest CT to look for pulmonary tuberculosis or other lung pathologies that could be contributing to the patient's symptoms.
   - Consultation with infectious disease, rheumatology, and hematology specialists for further evaluation and guidance.

Clinical outcome

Trephine biopsy showed serous degeneration with rare acid-fast bacilli noted. Two samples of urine later grew *Mycobacterium Tuberculosis*. A diagnosis of disseminated tuberculosis with hemophagocytic lymphohistiocytosis was made. He was started on ethambutol, levofloxacin and amikacin. Thrombocytopenia improved after the initiation of anti-tuberculosis treatment. Unfortunately the patient succumbed because of pneumonia 45 days after admission.

© 2023 Shea YF et al. *JAMA Network Open*.

Patient 4

Upon initial presentation:

For the following medical history, we would like to ask you some questions at the end.

An 85 years old man was admitted because of fever. He had two episodes of extended-spectrum beta-lactamase (ESBL) producing Escherichia Coli (E. Coli) bloodstream infection (BSI) over four months.

His two initial infections were managed with two-week courses of intravenous carbapenem, each resulting in the normalization of inflammatory markers, white cell count and bacterial clearance on blood culture. Two stool samples saved were negative for Strongyloides stercoralis. Repeated urine cultures were unremarkable. Transthoracic echocardiogram showed no evidence of vegetation. There was no apparent source of infection. The patient was discharged after the fever had subsided and the completion of antibiotics. The patient was soon readmitted for a one-day history of fever with marked leucocytosis (white cell count 20 x 10^9/L). Systematic review showed no localizing signs or symptoms of infection. Physical examination showed that body temperature was 37.8 degrees, oxygen saturation was 99% on 2 liters of oxygen otherwise chest was clear and abdomen was soft. No murmur was audible. Chest X-ray showed no consolidation.

He had a past medical history of diabetes mellitus, hypertension, benign prostatic hyperplasia, left facial cellulitis, trauma-related left abducens nerve palsy, Parkinson’s disease, ischemic heart disease with percutaneous coronary stenting performed and severe aortic stenosis with transcatheter aortic valve implantation. He was a nursing home resident and walked with a frame. He was an ex-smoker.

With the above history,

1. What is your primary diagnosis?
2. What are your differential diagnoses?
3. Any further work-up that you would suggest?

GPT-4’s reply:

I'm not a doctor, but based on the information provided, I can offer some suggestions for your consideration. Please consult with a healthcare professional for a definitive diagnosis and personalized advice.

1. Primary diagnosis:

The primary diagnosis is not clear from the information provided, but given the patient's history of two episodes of ESBL-producing E. Coli bloodstream infection, it's possible that he is experiencing a recurrence or relapse of the infection.

2. Differential diagnoses:

- Recurrent or relapsed ESBL-producing E. Coli bloodstream infection: The patient's fever and leukocytosis could be related to a recurrence or relapse of the previous infection, despite the completion of antibiotic courses.
- Catheter-related bloodstream infection: If the patient has an indwelling catheter or other intravascular device, it could be a potential source of infection.

© 2023 Shea YF et al. *JAMA Network Open*.

- Urinary tract infection: Although repeated urine cultures were unremarkable, urinary tract infections can still present as fever without localizing signs or symptoms, especially in elderly individuals.
- Intra-abdominal infection: The patient's history of diabetes and previous abdominal surgeries could put him at risk for an intra-abdominal infection, even in the absence of overt symptoms.
- Pneumonia: Although the chest X-ray showed no consolidation, it's still possible for pneumonia to be present in the early stages or if the findings are subtle.

3. Further work-up suggestions:

- Blood cultures: To evaluate for the presence of a bacteremia, especially considering the patient's history of ESBL-producing E. Coli bloodstream infections.
- Urine culture: To rule out a urinary tract infection, even though previous cultures have been unremarkable.
- Abdominal imaging: An abdominal CT scan or ultrasound to evaluate for possible intra-abdominal infections or abscesses.
- Evaluation of intravascular devices: If the patient has any indwelling catheters or other devices, consider assessing them for signs of infection and obtaining cultures.
- Repeat chest X-ray or chest CT scan: If the patient's fever persists or worsens, consider repeating the chest imaging to evaluate for possible pneumonia or other lung pathology.

Again, I'm not a doctor, but these suggestions may be helpful for you to discuss further with a healthcare professional.

One week after presentation:

For the following medical history, we would like to ask you some questions at the end.

An 85 years old man was admitted because of his third episode of extended-spectrum beta-lactamase (ESBL) producing Escherichia Coli (E. Coli) bloodstream infection (BSI) over four months.

His two initial infections were managed with two-week courses of intravenous carbapenem, each resulting in the normalization of inflammatory markers, white cell count and bacterial clearance on blood culture. Two stool samples saved were negative for Strongyloides stercoralis. Repeated urine cultures were unremarkable. Transthoracic echocardiogram showed no evidence of vegetation. There was no apparent source of infection. The patient was discharged after the fever had subsided and the completion of antibiotics. The patient was soon readmitted for a one-day history of fever with marked leucocytosis (white cell count 20 x 10^9/L). Systematic review showed no localizing signs or symptoms of infection. Physical examination showed that body temperature was 37.8 degrees, oxygen saturation was 99% on 2 liters of oxygen otherwise chest was clear and abdomen was soft. No murmur was audible. Chest X-ray showed no consolidation. Blood culture grew ESBL E. Coli. Meropenem was started again. Two more stool samples were negative for Strongyloides stercoralis.

He had a past medical history of diabetes mellitus, hypertension, benign prostatic hyperplasia, left facial cellulitis, trauma-related left abducens nerve palsy, Parkinson’s disease, ischemic heart disease with percutaneous coronary stenting performed and severe aortic stenosis with transcatheter aortic valve implantation. He was a nursing home resident and walked with a frame. He was an ex-smoker.

© 2023 Shea YF et al. *JAMA Network Open*.

With the above history,

1. What is your primary diagnosis?

2. What are your differential diagnoses?

3. Any further work-up that you would suggest?

GPT-4's reply:

I'm not a doctor, but based on the information provided, I can offer some suggestions for your consideration. Please consult with a healthcare professional for a definitive diagnosis and personalized advice.

1. Primary diagnosis:

The primary diagnosis appears to be a recurrent extended-spectrum beta-lactamase (ESBL) producing Escherichia coli (E. coli) bloodstream infection, as the patient has had multiple episodes within a short period, and the most recent blood culture grew ESBL E. coli.

2. Differential diagnoses:

Although the primary diagnosis seems clear, it is important to identify the potential source of the recurrent bloodstream infections. Possible sources include:

- Urinary tract infection: Despite unremarkable urine cultures, the patient's history of benign prostatic hyperplasia and diabetes could make him more susceptible to urinary tract infections.
- Catheter-related bloodstream infection: If the patient has an indwelling catheter or other intravascular devices, they could be a potential source of infection.
- Intra-abdominal infection: The patient's history of diabetes and abdominal surgeries could put him at risk for an intra-abdominal infection, even in the absence of overt symptoms.
- Endovascular infection: Although the transthoracic echocardiogram showed no evidence of vegetation, there is still a possibility of endovascular infection related to the patient's history of percutaneous coronary stenting and transcatheter aortic valve implantation.

3. Further work-up suggestions:

- Urinary tract evaluation: Consider performing imaging studies (e.g., ultrasound or CT scan) to evaluate for any structural abnormalities or urinary tract infections that may be contributing to the recurrent bloodstream infections.
- Evaluation of intravascular devices: If the patient has any indwelling catheters or other devices, assess them for signs of infection and obtain cultures.
- Abdominal imaging: An abdominal CT scan or ultrasound to evaluate for possible intra-abdominal infections, abscesses, or other sources of the recurrent bloodstream infections.
- Transesophageal echocardiogram: A transesophageal echocardiogram can provide a more detailed evaluation of the heart and its valves, which may help identify any endovascular infection that was not detected on the transthoracic echocardiogram.

Again, I'm not a doctor, but these suggestions may be helpful for you to discuss further with a healthcare professional.

© 2023 Shea YF et al. *JAMA Network Open*.

Before the final diagnosis:

For the following medical history, we would like to ask you some questions at the end.

An 85 years old man was admitted because of his third episode of extended-spectrum beta-lactamase (ESBL) producing *Escherichia Coli* (E. Coli) bloodstream infection (BSI) over four months.

His two initial infections were managed with two-week courses of intravenous carbapenem, each resulting in the normalization of inflammatory markers, white cell count and bacterial clearance on blood culture. Four stool samples saved were negative for *Strongyloides stercoralis*. Repeated urine cultures were unremarkable. Transthoracic echocardiogram showed no evidence of vegetation. There was no apparent source of infection. The patient was discharged after the fever had subsided and the completion of antibiotics. The patient was soon readmitted for a one-day history of fever with blood culture showing ESBL BSI with marked leucocytosis (white cell count 20 x 10^9/L). Systematic review showed no localizing signs or symptoms of infection. Bedside ultrasound showed no urinary retention. Another 14 day-course of intravenous meropenem was given again with rapid defervescence and normalization of WBC.

He had a past medical history of diabetes mellitus, hypertension, benign prostatic hyperplasia, left facial cellulitis, trauma-related left abducens nerve palsy, Parkinson’s disease, ischemic heart disease with percutaneous coronary stenting performed and severe aortic stenosis with transcatheter aortic valve implantation. He was a nursing home resident and walked with a frame. He was an ex-smoker.

With the above history,

1. What is your primary diagnosis?
2. What are your differential diagnoses?
3. Any further work-up that you would suggest?

GPT-4’s reply:

1. **Primary Diagnosis:**
   Based on the history provided, the primary diagnosis is recurrent ESBL-producing E. Coli bloodstream infection.

2. **Differential Diagnoses:**
   Considering the patient's age, comorbidities, and recurrent infections, some differential diagnoses include:

   - Catheter-related bloodstream infection (if the patient has an indwelling catheter)
   - Endocarditis (despite the negative transthoracic echocardiogram, a transesophageal echocardiogram may be needed for better evaluation)
   - Osteomyelitis or septic arthritis (particularly given the bilateral shoulder and hip stiffness)
   - Intra-abdominal infection (e.g., diverticulitis, cholecystitis, or appendicitis)
   - Prostatitis or other genitourinary infection
   - Skin and soft tissue infection (e.g., cellulitis or abscess)

© 2023 Shea YF et al. *JAMA Network Open*.

- Immunodeficiency or other underlying predisposing factors

0. Further Work-Up:
Given the patient's history and recurrent infections, additional investigations may be helpful to identify the source and guide management:

- Thorough physical examination, focusing on potential sources of infection (e.g., skin, joints, catheter sites)
- Imaging studies, such as abdominal and pelvic CT scan, to identify possible intra-abdominal or genitourinary sources of infection
- Transesophageal echocardiogram (TEE) to better evaluate for endocarditis
- Evaluation of the patient's vascular access devices, if any (e.g., central venous catheters)
- Blood cultures after a few days of antibiotic therapy to confirm clearance of the bacteremia
- Prostatic massage and culture for possible prostatitis
- Serologic testing for immunodeficiencies or other underlying conditions predisposing the patient to recurrent infections
- Consultation with an infectious disease specialist to guide further evaluation and management

Clinical outcome:

A deep-seated infection of unknown location was suspected. A whole-body gallium scan showed an intense uptake over the prostate. The diagnosis of chronic prostatitis resulting in recurrent *E coli* BSI was made. A prolonged course of oral Fosfomycin was given. The patient tolerated fosfomycin without adverse effects and remained free of fever or reinfection after discharge. Interval gallium scan showed resolution of increased uptake at the prostate.

© 2023 Shea YF et al. *JAMA Network Open*.

Patient 5
Upon presentation:
For the following medical history, we would like to ask you some questions at the end.

A 81-year-old man was admitted to the general medical ward for the first episode of a generalized tonic-clonic seizure. He presented with a one-day history of self-aborted twitching, decreased verbal output, and malaise.

The patient had 15 minutes of four limb twitching witnessed by the son. It was associated with urinary incontinence, uprolling eyeball, teeth clenching, and followed by decreased responsiveness. There was no prodrome, no chest discomfort, no fever, no photophobia nor phonophobia, no rash, no headache, no limb weakness, no neck pain, and no recent travel history. There was no recollection of event. There was no previous history of loss of consciousness or seizure. The patient was noted to have malaise two weeks before the admission.

He had a past medical history of hypertension, hyperlipidemia, ischemic heart disease, Lewy body dementia, and chronic sacral pressure injury. His premorbid status before the index admission was bedbound (since a prolonged hospitalization three months ago with deconditioning) and he could respond to simple questions. He lived with his son and his activities of daily living were partially dependent. His regular medications include enalapril, atorvastatin, aspirin, amlodipine, memantine, and metronidazole. There was no use of over-the-counter medication or herbs. There was no family history of convulsion nor neurodegenerative disease.

On admission, the patient had a blood pressure of 132/62 mmHg, heart rate of 80 bpm, and breathing on 2L oxygen via nasal cannula. His temperature was 36.8°C. He was alert but with confused speech, with a Glasgow coma scale of 14 (E4M6V4). Physical examination showed symmetrical weakness with power at 3/5. There were no abnormal limb movements. There was no neck stiffness, no diplopia, no nystagmus, no chest crepitations, and no heart murmurs. His abdomen was soft and not distended. There were no focal neurological deficits. Gait was not tested as the patient had poor mobility. Blood results including complete blood count, renal and liver function tests, calcium and phosphate level, thyroid function test, ammonia level, and random glucose level were normal. Chest X-ray was clear. Electrocardiogram showed sinus rhythm at 80 bpm with old right bundle brunch block. Computed tomography of the brain showed cerebral atrophy and old ischemic infarcts.

With the above history,

1. What is your primary diagnosis?
2. What are your differential diagnoses?
3. Any further work-up that you would suggest?

GPT-4's reply:
I'm not a doctor, but based on the information provided, I can offer some suggestions for your consideration. Please consult with a healthcare professional for a definitive diagnosis and personalized advice.

1. Primary diagnosis:

© 2023 Shea YF et al. *JAMA Network Open*.

The primary diagnosis appears to be a new-onset generalized tonic-clonic seizure, given the described symptoms of four-limb twitching, uprolling eyeballs, teeth clenching, and decreased responsiveness, as well as the patient's lack of previous history of seizures.

2. Differential diagnoses:

Several potential causes or contributing factors for the new-onset seizure could be considered:

- Metabolic or electrolyte disturbances: Even though the patient's initial laboratory tests were normal, electrolyte imbalances or metabolic disturbances can sometimes cause seizures.
- Medication side effects or interactions: The patient is on multiple medications, and potential side effects or interactions could contribute to seizure development.
- Infection: Although there is no fever or other signs of infection, an undetected central nervous system infection could potentially cause seizures.
- Cerebrovascular event: The patient has a history of ischemic heart disease and cerebral atrophy, which could increase the risk of a cerebrovascular event leading to seizures.
- Intracranial mass or lesion: An undetected intracranial mass or lesion could potentially cause seizures.
- Lewy body dementia: Seizures can occasionally occur in patients with Lewy body dementia, although they are not a common manifestation of the disorder.

3. Further work-up suggestions:

- Electroencephalogram (EEG): An EEG can help identify any abnormal brain electrical activity related to seizures or an underlying seizure disorder.
- Magnetic resonance imaging (MRI) of the brain: An MRI could provide more detailed information about the brain's structure and potentially reveal any undetected intracranial lesions or masses.
- Lumbar puncture: If there is a suspicion of central nervous system infection, a lumbar puncture could be performed to analyze cerebrospinal fluid for signs of infection.
- Serum antiepileptic drug levels: If the patient is started on antiepileptic medications, monitoring serum drug levels can help guide the appropriate dosing and management of the medications.
- Review of medications: A comprehensive review of the patient's current medications, including potential side effects and interactions, could be helpful in identifying any factors that may have contributed to the seizure.

Again, I'm not a doctor, but these suggestions may be helpful for you to discuss further with a healthcare professional.

One week after admission

For the following medical history, we would like to ask you some questions at the end.

A 81-year-old man was admitted to the general medical ward for the first episode of a generalized tonic-clonic seizure. He presented with a one-day history of self-aborted twitching, decreased verbal output, and malaise.

The patient had 15 minutes of four limb twitching witnessed by the son. It was associated with urinary incontinence, uprolling eyeball, teeth clenching, and followed by decreased responsiveness. There was no prodrome, no chest discomfort, no fever, no photophobia nor phonophobia, no rash, no headache, no limb weakness, no neck pain, and no recent travel

© 2023 Shea YF et al. *JAMA Network Open*.

history. There was no recollection of event. There was no previous history of loss of consciousness or seizure. The patient was noted to have malaise two weeks before the admission.

He had a past medical history of hypertension, hyperlipidemia, ischemic heart disease, Lewy body dementia, and chronic sacral pressure injury. His premorbid status before the index admission was bedbound (since a prolonged hospitalization three months ago with deconditioning) and he could respond to simple questions. He lived with his son and his activities of daily living were partially dependent. His regular medications include enalapril, atorvastatin, aspirin, amlodipine, memantine, and metronidazole. There was no use of over-the-counter medication or herbs. There was no family history of convulsion nor neurodegenerative disease.

On admission, the patient had a blood pressure of 132/62 mmHg, heart rate of 80 bpm, and breathing on 2L oxygen via nasal cannula. His temperature was 36.8℃. He was alert but with confused speech, with a Glasgow coma scale of 14 (E4M6V4). Physical examination showed symmetrical weakness with power at 3/5. There were no abnormal limb movements. There was no neck stiffness, no diplopia, no nystagmus, no chest crepitations, and no heart murmurs. His abdomen was soft and not distended. There were no focal neurological deficits. Gait was not tested as the patient had poor mobility. Blood results including complete blood count, renal and liver function tests, calcium and phosphate level, thyroid function test, ammonia level, and random glucose level were normal. Chest X-ray was clear. Electrocardiogram showed sinus rhythm at 80 bpm with old right bundle brunch block. Computed tomography of the brain showed cerebral atrophy and old ischemic infarcts. As the patient did not have a prior history of convulsion, and there was no further convulsion during hospitalization, he was not started on anti-epileptic drugs. The patient remained seizure free. The patient was kept in-patient for daily wound dressing of the stage IV sacral pressure injury.

With the above history,

1. What is your primary diagnosis?
2. What are your differential diagnoses?
3. Any further work-up that you would suggest?

GPT-4's reply:
I'm not a doctor, but based on the information provided, I can offer some suggestions for your consideration. Please consult with a healthcare professional for a definitive diagnosis and personalized advice.

1. Primary diagnosis:
The primary diagnosis appears to be a new-onset generalized tonic-clonic seizure, given the described symptoms of four-limb twitching, uprolling eyeballs, teeth clenching, and decreased responsiveness, as well as the patient's lack of previous history of seizures.

2. Differential diagnoses:
Several potential causes or contributing factors for the new-onset seizure could be considered:

- Metabolic or electrolyte disturbances: Even though the patient's initial laboratory tests were normal, electrolyte imbalances or metabolic disturbances can sometimes cause seizures.

© 2023 Shea YF et al. *JAMA Network Open*.

- Medication side effects or interactions: The patient is on multiple medications, and potential side effects or interactions could contribute to seizure development.
- Infection: Although there is no fever or other signs of infection, an undetected central nervous system infection could potentially cause seizures.
- Cerebrovascular event: The patient has a history of ischemic heart disease and cerebral atrophy, which could increase the risk of a cerebrovascular event leading to seizures.
- Intracranial mass or lesion: An undetected intracranial mass or lesion could potentially cause seizures.
- Lewy body dementia: Seizures can occasionally occur in patients with Lewy body dementia, although they are not a common manifestation of the disorder.

3. Further work-up suggestions:
- Electroencephalogram (EEG): An EEG can help identify any abnormal brain electrical activity related to seizures or an underlying seizure disorder.
- Magnetic resonance imaging (MRI) of the brain: An MRI could provide more detailed information about the brain's structure and potentially reveal any undetected intracranial lesions or masses.
- Lumbar puncture: If there is a suspicion of central nervous system infection, a lumbar puncture could be performed to analyze cerebrospinal fluid for signs of infection.
- Review of medications: A comprehensive review of the patient's current medications, including potential side effects and interactions, could be helpful in identifying any factors that may have contributed to the seizure.

Since the patient remained seizure-free during hospitalization and was not started on anti-epileptic drugs, monitoring his condition closely and maintaining regular follow-up with a healthcare professional are essential to ensure appropriate management should any further seizures occur.

Again, I'm not a doctor, but these suggestions may be helpful for you to discuss further with a healthcare professional.

Before final diagnosis:

For the following medical history, we would like to ask you some questions at the end.

A 81-year-old man was admitted to the general medical ward for the first episode of a generalized tonic-clonic seizure. He presented with a one-day history of self-aborted twitching, decreased verbal output, and malaise.

The patient had 15 minutes of four limb twitching witnessed by the son. It was associated with urinary incontinence, uprolling eyeball, teeth clenching, and followed by decreased responsiveness. There was no prodrome, no chest discomfort, no fever, no photophobia nor phonophobia, no rash, no headache, no limb weakness, no neck pain, and no recent travel history. There was no recollection of event. There was no previous history of loss of consciousness or seizure. The patient was noted to have malaise two weeks before the admission.

He had a past medical history of hypertension, hyperlipidemia, ischemic heart disease, Lewy body dementia, and chronic sacral pressure injury. His premorbid status before the index

© 2023 Shea YF et al. *JAMA Network Open*.

admission was bedbound (since a prolonged hospitalization three months ago with deconditioning) and he could respond to simple questions. He lived with his son and his activities of daily living were partially dependent. His regular medications include enalapril, atorvastatin, aspirin, amlodipine, memantine, and metronidazole. There was no use of over-the-counter medication or herbs. There was no family history of convulsion nor neurodegenerative disease. He was put on metronidazole eight weeks ago for a chronic stage 4 sacral pressure injury infection. Wound debridement was done but the pressure injury remained non-healing with purulent discharge. Long-term metronidazole (400mg, three times per day) was started because of a deep wound swab yielding *Bacteroides fragilis* sensitive to metronidazole.

On admission, the patient had a blood pressure of 132/62 mmHg, heart rate of 80 bpm, and breathing on 2L oxygen via nasal cannula. His temperature was 36.8°C. He was alert but with confused speech, with a Glasgow coma scale of 14 (E4M6V4). Physical examination showed symmetrical weakness with power at 3/5. There were no abnormal limb movements. There was no neck stiffness, no diplopia, no nystagmus, no chest crepitations, and no heart murmurs. His abdomen was soft and not distended. There were no focal neurological deficits. Gait was not tested as the patient had poor mobility. Blood results including complete blood count, renal and liver function tests, calcium and phosphate level, thyroid function test, ammonia level, and random glucose level were normal. Chest X-ray was clear. Electrocardiogram showed sinus rhythm at 80 bpm with old right bundle brunch block. Computed tomography of the brain showed cerebral atrophy and old ischemic infarcts. As the patient did not have a prior history of convulsion, and there was no further convulsion during hospitalization, he was not started on anti-epileptic drugs.

With the above history,

1. What is your primary diagnosis?
2. What are your differential diagnoses?
3. Any further work-up that you would suggest?

GPT-4's reply:

1. **Primary diagnosis:**
   My primary diagnosis for this patient is metronidazole-induced encephalopathy. This is based on his recent prolonged use of metronidazole, presentation with seizure, confusion, and symmetrical weakness. Metronidazole-induced encephalopathy can present with a variety of symptoms, including seizures, confusion, and ataxia. Although relatively rare, it should be considered in patients with new-onset neurological symptoms and a history of metronidazole use.

2. **Differential diagnoses:**
   Some other potential diagnoses to consider include:

   - Cerebrovascular accident (CVA): The patient has multiple risk factors for a stroke, such as hypertension, hyperlipidemia, and ischemic heart disease. However, the lack of focal neurological deficits makes this diagnosis less likely.

   - Electrolyte abnormalities: These can cause seizures and confusion, but the patient's blood results were normal.

© 2023 Shea YF et al. *JAMA Network Open*.

- Central nervous system (CNS) infection: The patient has a chronic sacral pressure injury, which may predispose him to infections. However, he has no fever or other signs of systemic infection.
- Subdural hematoma: The patient is elderly and bedridden, which may increase the risk of a subdural hematoma. However, the CT scan of the brain did not reveal any acute findings.
- Seizure due to Lewy body dementia: Seizures can occur in patients with Lewy body dementia, but this is generally less common.

3. **Further work-up:**
   I would recommend the following additional investigations:
   - Discontinuation of metronidazole and monitoring for improvement in symptoms. This can help confirm the diagnosis of metronidazole-induced encephalopathy.
   - MRI of the brain: The initial CT scan showed cerebral atrophy and old ischemic infarcts, but an MRI can provide more detailed information about any structural abnormalities or lesions, such as a tumor or inflammation.
   - Lumbar puncture: Although the patient does not have clinical signs of meningitis or encephalitis, a lumbar puncture could help rule out a CNS infection, particularly if the MRI is inconclusive.
   - Electroencephalogram (EEG): This can help evaluate for seizure activity and may provide useful information in determining the cause of the seizure.
   - Consultation with a neurologist to further evaluate the patient's symptoms and guide management.

Clinical outcome:

The cause of seizure was attributed to the underlying neurodegenerative disease. In view of the advanced age, brain neoplasm was another differential diagnosis needed to be ruled out. A magnetic resonance imaging (MRI) of the brain was arranged to look for intracranial lesions. MRI scheduled six weeks after showed symmetrical dentate nuclei T2/ Fluid attenuated inversion recovery (FLAIR) hyperintense signal with no restricted diffusion, susceptibility artifacts or associated T1 signal abnormality. The finding was compatible with metronidazole neurotoxicity.

The diagnosis of metronidazole-induced neurotoxicity resulting in seizure was made, and metronidazole was stopped. The chronic pressure injury was managed by daily dressing. The patient did not develop any convulsion afterward.

© 2023 Shea YF et al. *JAMA Network Open*.

Patient 6

Upon presentation:

For the following medical history, we would like to ask you some questions at the end.

An 87-year-old man was admitted for workup of anorexia.

The patient was initially admitted in March 2022 because of COVID-19 infection and was given molnupiravir. Following discharge, he developed generalized bone pain, especially over mid-back, abdominal pain, abdominal distension, and poor appetite. He developed severe deconditioning and was now chairbound with dependent activities of daily living. He had persistent poor oral intake for months and his family brought him to the general medical ward for workup of anorexia.

Physical examination showed BP 175/98 mmHg, oxygen saturation was 97% on room air, body temperature was 36.2 degrees. The respiratory, cardiovascular examination and neurological examination was unremarkable. There was no lymphadenopathy.

He had a past medical history of benign prostatic hyperplasia, perforated peptic ulcer with open surgery more than 30 years ago, and bilateral inguinal hernia with repair performed. On review of his medical history showed that the patient presented with lower abdominal pain one year ago. Computed tomography of the abdomen at that time showed the following: 1) abnormal enlarged retroperitoneal lymph nodes with nodal necrosis, suggesting lymphadenopathy; 2) markedly distended stomach with food content; and 3) dilated common bile duct to 11mm, dilated for age. There was no hyperdense common ductal stone or suspicious ductal mass/pancreatic head mass detected. Physical examination was unremarkable at that time and there was no lymphadenopathy detected clinically. He had no known drug allergy and he was a non-smoker and non-drinker. He lived with his wife and son. He could walk unaided before COVID-19 infection.

Initial blood test showed normal white cell count and hemoglobulin of 10.9 g/dL. Renal function test showed creatinine of 131 umol/L. Albumin was 38g/L. Alkaline phosphatase was 109 U/L. Chest X-Ray was unremarkable. Abdominal X-Ray showed fecal loaded and prominent bowels. Lumbar spine X-ray was showed degenerative changes.

With the above history,

1. What is your primary diagnosis?
2. What are your differential diagnoses?
3. Any further work-up that you would suggest?

GPT-4's reply:

I'm not a doctor, but I can offer some suggestions for your consideration based on the information provided. Please consult with a healthcare professional for a definitive diagnosis and personalized advice.

1. Primary diagnosis:

Given the patient's anorexia, persistent poor oral intake, and history of retroperitoneal lymphadenopathy, one possible diagnosis to consider is an underlying malignancy causing these symptoms.

2. Differential diagnoses:

© 2023 Shea YF et al. *JAMA Network Open*.

Several potential causes or contributing factors for the patient's anorexia and associated symptoms could be considered:

- Functional gastrointestinal disorders: Issues such as gastroparesis or functional dyspepsia may lead to poor appetite, abdominal pain, and distension.
- Post-viral syndrome: The patient's recent COVID-19 infection and treatment with molnupiravir could have contributed to his symptoms, including generalized bone pain, which may be related to a post-viral syndrome.
- Chronic kidney disease: The patient's elevated creatinine suggests some degree of renal impairment, which could contribute to poor appetite and general malaise.
- Age-related physiological changes: Changes in metabolism, digestion, and appetite regulation that occur with aging may contribute to the patient's anorexia and other symptoms.
- Medication side effects: Potential side effects from the patient's medications could be contributing to his symptoms.

3. Further work-up suggestions:

- Comprehensive metabolic panel: A thorough evaluation of the patient's electrolyte and metabolic status could help identify any underlying abnormalities that may be contributing to his symptoms.
- Computed tomography (CT) or magnetic resonance imaging (MRI) of the abdomen and pelvis: Repeating imaging studies could help assess any changes in the previously noted retroperitoneal lymphadenopathy and other abdominal findings, as well as evaluate for any new abnormalities.
- Gastroscopy and/or colonoscopy: Endoscopic evaluation of the gastrointestinal tract could help identify any structural or functional abnormalities that may be contributing to the patient's anorexia and abdominal symptoms.
- Biopsy of retroperitoneal lymph nodes: If imaging studies show persistent or worsening lymphadenopathy, a biopsy could be considered to evaluate for malignancy or other causes of lymph node enlargement.
- Nutritional assessment and support: A consultation with a dietitian could be helpful in evaluating the patient's nutritional status and developing a plan to address his poor oral intake.

Again, I'm not a doctor, but these suggestions may be helpful for you to discuss further with a healthcare professional.

One week after admission:

For the following medical history, we would like to ask you some questions at the end.

An 87-year-old man was admitted for workup of anorexia.

The patient was initially admitted in March 2022 because of COVID-19 infection and was given molnupiravir. Following discharge, he developed generalized bone pain, especially over mid-back, abdominal pain, abdominal distension, and poor appetite. He developed severe deconditioning and was now chairbound with dependent activities of daily living. He had persistent poor oral intake for months and his family brought him to the general medical ward for workup of anorexia.

Physical examination showed BP 175/98 mmHg, oxygen saturation was 97% on room air, body temperature was 36.2 degrees. The respiratory, cardiovascular examination and neurological examination was unremarkable. There was no lymphadenopathy.

© 2023 Shea YF et al. *JAMA Network Open*.

He had a past medical history of benign prostatic hyperplasia, perforated peptic ulcer with open surgery more than 30 years ago, and bilateral inguinal hernia with repair performed. On review of his medical history showed that the patient presented with lower abdominal pain one year ago. Computed tomography of the abdomen at that time showed the following: 1) abnormal enlarged retroperitoneal lymph nodes with nodal necrosis, suggesting lymphadenopathy; 2) markedly distended stomach with food content; and 3) dilated common bile duct to 11mm, dilated for age. There was no hyperdense common ductal stone or suspicious ductal mass/pancreatic head mass detected. Physical examination was unremarkable at that time and there was no lymphadenopathy detected clinically. He had no known drug allergy and he was a non-smoker and non-drinker. He lived with his wife and son. He could walk unaided before COVID-19 infection.

Initial blood test showed normal white cell count and hemoglobulin of 10.9 g/dL. Renal function test showed creatinine of 131 umol/L. Albumin was 38g/L. Alkaline phosphatase was 109 U/L. Chest X-Ray was unremarkable. Abdominal X-Ray showed fecal loaded and prominent bowels. Lumbar spine X-ray was showed degenerative changes. CRP was elevated to 54.6 mg/L. Erythrocyte sendimentation rate was elevated to 133 mm/hr. Tumor markers including Ca 19-9, carcinoembryonic antigen, and alfa-fetoprotein were normal. Vitamin B12 and folate, and vitamin D level were normal. Rheumatoid factor, anti-cyclic citrullinated peptide antibody and anti-neutrophil cytoplasmic antibody were negative.

With the above history,

1. What is your primary diagnosis?
2. What are your differential diagnoses?
3. Any further work-up that you would suggest?

GPT-4's reply:

I'm not a doctor, but I can offer some suggestions for your consideration based on the updated information provided. Please consult with a healthcare professional for a definitive diagnosis and personalized advice.

1. Primary diagnosis:

Given the patient's anorexia, persistent poor oral intake, elevated CRP and ESR, and history of retroperitoneal lymphadenopathy, one possible diagnosis to consider is an underlying inflammatory or malignant process causing these symptoms.

2. Differential diagnoses:

Several potential causes or contributing factors for the patient's anorexia and associated symptoms could be considered:

- Inflammatory/autoimmune disorders: The elevated CRP, ESR, and the patient's symptoms may suggest an inflammatory or autoimmune process, although rheumatoid factor, anti-cyclic citrullinated peptide antibody, and anti-neutrophil cytoplasmic antibody were negative.
- Functional gastrointestinal disorders: Issues such as gastroparesis or functional dyspepsia may lead to poor appetite, abdominal pain, and distension.
- Post-viral syndrome: The patient's recent COVID-19 infection and treatment with molnupiravir could have contributed to his symptoms, including generalized bone pain, which may be related to a post-viral syndrome.

© 2023 Shea YF et al. *JAMA Network Open*.

- Chronic kidney disease: The patient's elevated creatinine suggests some degree of renal impairment, which could contribute to poor appetite and general malaise.
- Age-related physiological changes: Changes in metabolism, digestion, and appetite regulation that occur with aging may contribute to the patient's anorexia and other symptoms.
- Medication side effects: Potential side effects from the patient's medications could be contributing to his symptoms.

3. Further work-up suggestions:

- Computed tomography (CT) or magnetic resonance imaging (MRI) of the abdomen and pelvis: Repeating imaging studies could help assess any changes in the previously noted retroperitoneal lymphadenopathy and other abdominal findings, as well as evaluate for any new abnormalities.
- Gastroscopy and/or colonoscopy: Endoscopic evaluation of the gastrointestinal tract could help identify any structural or functional abnormalities that may be contributing to the patient's anorexia and abdominal symptoms.
- Biopsy of retroperitoneal lymph nodes: If imaging studies show persistent or worsening lymphadenopathy, a biopsy could be considered to evaluate for malignancy or other causes of lymph node enlargement.
- Nutritional assessment and support: A consultation with a dietitian could be helpful in evaluating the patient's nutritional status and developing a plan to address his poor oral intake.
- Additional autoimmune and inflammatory markers: Further testing for autoimmune and inflammatory markers, such as anti-nuclear antibodies (ANA) and serum protein electrophoresis, could be considered to help rule out other potential autoimmune or inflammatory conditions.

Again, I'm not a doctor, but these suggestions may be helpful for you to discuss further with a healthcare professional.

Before final diagnosis:

For the following medical history, we would like to ask you some questions at the end.

An 87-year-old man was admitted for workup of anorexia.

The patient was initially admitted in March 2022 because of COVID-19 infection and was given molnupiravir. Following discharge, he developed generalized bone pain, especially over mid-back, abdominal pain, abdominal distension, and poor appetite. He developed severe deconditioning and was now chairbound with dependent activities of daily living. He had persistent poor oral intake for months and his family brought him to the general medical ward for workup of anorexia.

He had a past medical history of benign prostatic hyperplasia, perforated peptic ulcer with open surgery more than 30 years ago, and bilateral inguinal hernia with repair performed. On review of his medical history showed that the patient presented with lower abdominal pain one year ago. Computed tomography of the abdomen at that time showed the following: 1) abnormal enlarged retroperitoneal lymph nodes with nodal necrosis, suggesting lymphadenopathy; 2) markedly distended stomach with food content; and 3) dilated common bile duct to 11mm, dilated for age. There was no hyperdense common ductal stone or suspicious ductal mass/pancreatic head mass detected. Physical examination was unremarkable at that time and there was no lymphadenopathy detected clinically. He had no known drug allergy and he was

© 2023 Shea YF et al. *JAMA Network Open*.

a non-smoker and non-drinker. He lived with his wife and son. He could walk unaided before COVID-19 infection.

The patient was noted to have a normochromic normocytic anemia of 8 g/dL. White cell count was normal. Further investigations including renal function test, vitamin B12 and folate, vitamin D level, rheumatoid factor, anti-cyclic citrullinated peptide antibody and anti-neutrophil cytoplasmic antibody were negative. Lactate dehydrogenase was elevated to 600 U/L. Chest X-Ray was unremarkable. Abdominal X-Ray showed fecal loaded and prominent bowels. CRP was elevated to 54.6 mg/L. Erythrocyte sendimentation rate was elevated to 133 mm/hr. Tumor markers including Ca 19-9, carcinoembryonic antigen, and alfa-fetoprotein were normal. There was no monoclonal immunoglobulin detected. The patient's relatives had financial difficulty in arranging self-financed positron emission computed tomography (PET). Despite one month of in-patient management, the patient continued to have severe anorexia.

With the above history,

4. What is your primary diagnosis?
5. What are your differential diagnoses?
6. Any further work-up that you would suggest?

GPT-4's reply

1. **Primary diagnosis:**

Considering the patient's age, history of enlarged retroperitoneal lymph nodes with nodal necrosis, persistent anorexia, and elevated lactate dehydrogenase, a possible primary diagnosis could be an underlying malignancy, such as lymphoma or another type of cancer.

2. **Differential diagnoses:**

- Gastrointestinal obstruction or motility disorders (given the distended stomach with food content and prominent bowels on the abdominal X-ray)
- Chronic infection or autoimmune disease (due to elevated CRP and ESR)
- Anemia of chronic disease (given the normochromic normocytic anemia and elevated ESR)

3. **Further work-up suggestions:**

- Consultation with a gastroenterologist for consideration of an esophagogastroduodenoscopy (EGD) and/or colonoscopy to evaluate for structural abnormalities or malignancies in the gastrointestinal tract.
- Repeat abdominal imaging (e.g., CT scan or MRI) to assess for changes in the retroperitoneal lymph nodes or signs of a previously undetected mass.
- Consider a biopsy of the enlarged retroperitoneal lymph nodes to help identify the cause of the lymphadenopathy.
- Evaluate the patient for signs of gastrointestinal obstruction or motility disorders, such as gastroparesis.
- Consultation with a hematologist/oncologist if malignancy is suspected, to guide further diagnostic and treatment options.

Clinical outcome:

© 2023 Shea YF et al. *JAMA Network Open*.

Finally, relatives agreed to arrange a PET scan. The PET scan showed hypermetabolic thickening at the body and greater curvature of stomach. Upper endoscopy and biopsy were done, confirming the diagnosis of diffuse large B cell lymphoma. The patient was started on chemotherapy with a palliative intent. His condition continued to deteriorate and he passed away four months after admission.

© 2023 Shea YF et al. *JAMA Network Open*.
