# Supplementary material for: Use of GPT-4 to Analyze Medical Records of Patients With Extensive Investigations and Delayed Diagnosis
Source: JAMA Netw Open. 2023 Aug 14;6(8):e2325000. doi: 10.1001/jamanetworkopen.2023.25000 (PMC10425828; doi:10.1001/jamanetworkopen.2023.25000)
Supplement: Supplement 2. — Data Sharing Statement [file jamanetwopen-e2325000-s002.pdf]

Data Sharing Statement

Shea. Use of GPT-4 to Analyze Medical Records of Patients With Extensive Investigations and Delayed Diagnosis. *JAMA Netw Open*. Published August 14, 2023.
doi:10.1001/jamanetworkopen.2023.25000

Data
Data available: Yes
Data types: Deidentified participant data
How to access data: Provide complete email address
When available: With publication

Supporting Documents
Document types: None

Additional Information
Who can access the data: anyone requesting the data
Types of analyses: for any purpose
Mechanisms of data availability: with a signed data access agreement
